# Typhoid Fever among Admitted Pediatric Patients in a Tertiary Care Center: A Descriptive Cross-sectional Study

**DOI:** 10.31729/jnma.6044

**Published:** 2021-09-30

**Authors:** Gajendra Prasad Rauniyar, Shrawanti Bhattacharya, Kumud Chapagain, Gauri Shankar Shah, Basundha Khanal

**Affiliations:** 1 Department of Clinical Pharmacology and Therapeutics, B.P. Koirala Institute of Health Sciences, Dharan, Nepal; 2 Department of Pediatrics & Adolescent Medicine, B.P. Koirala Institute of Health Sciences, Dharan, Nepal; 3 Department of Microbiology, B.P. Koirala Institute of Health Sciences, Dharan, Nepal

**Keywords:** *antibiotic*, *enteric fever*, *typhoid*

## Abstract

**Introduction:**

Typhoid fever, an acute systemic febrile illness caused by Salmonella Typhi & Paratyphi, is an important public health problem in developing countries. It requires frequent observation regarding proper diagnostic protocol and treatment practices. The aim of the study is to find the prevalence of typhoid fever among admitted pediatric patients in a tertiary care center.

**Methods:**

This is a descriptive cross-sectional study conducted among the admitted patients of pediatric and adolescent medicine of a tertiary care center from August 2016 to May 2018 after obtaining ethical clearance (IRC/609/015). Convenience sampling was used and data was analyzed using the Statistical Package of Social version 11.5. Point estimate at 95% Confidence Interval was calculated along with frequency and proportion for binary data.

**Results:**

Among 7450 patients, 151 (2.03%) at 95% Confidence Interval (1.71-2.35) patients were diagnosed with enteric fever of which 85 (56.29%) were male and 66 (43.71%) were female. Common symptoms were fever 151 (100%), and abdominal pain 94 (62.25%). Azithromycin 54 (38.03%) was the most common antibiotic received before presenting to hospital and ceftriaxone 151 (100%) was prescribed to all the patients after admission. Two-third of the patients (96/151) was hospitalized for at least 6 days, with the longest hospital stay of 14 days and shortest of 3 days.

**Conclusions:**

Occurrence rate of Typhoid Fever was similar to other studies. Antibiotic susceptibility could not be well established; further surveillance on typhoid fever and the antimicrobial susceptibility pattern is recommended.

## INTRODUCTION

Typhoid (enteric) fever (TF), a systemic infection caused by the bacterium Salmonella Typhi and Paratyphi, is an important public health problem in developing countries. WHO estimates between 11 and 21 million cases and 128 000 to 161 000 typhoid-related deaths occur annually worldwide.^1^ In Nepal, TF is endemic with seasonal outbreaks.^2^ The outbreak is more common during monsoon and is usually due to contamination of food and drinking water.

TF in some patients can develop life threatening complications. It remains a public health concern and the incidence is highest in children with higher rates of hospitalization.^3^ Antimicrobial agents are the definitive therapy in order to prevent the complications associated with TF.^3^ Appropriate and timely antibiotic administration reduces the durations of fever from 3-4 weeks to 3-4 days.^4^

In this study we aim to find the prevalence of typhoid fever among admitted pediatric patients of a tertiary care center in Eastern Nepal.

## METHODS

A descriptive cross-sectional study was conducted among the admitted patients of pediatric and adolescent medicine ward in B.P. Koirala Institute of Health Sciences (BPKIHS), Dharan, Nepal from August 2016 to May 2018. Ethical approval was obtained from the Institutional Review Committee of B.P. Koirala Institute of Health Sciences (IRC). Convenience sampling technique was used and the sample size was calculated using the formula,

n = Z^2^ × p × q / e^2^

  = 1.96^2^ × 0.50 × 0.50 / (0.02)^2^

  = 2401

Where,

n = minimum required sample size
Z = 1.96 at 95% confidence interval
p = prevalence taken as 50%
q = 1-p
e = margin of error, 2%

Since convenience sampling was used after doubling the sample size, the sample size becomes 4802, however we took a sample size of 7450.

Data was collected from all the pediatric patients (0-18 years) admitted during the study period with fever or history of fever with clinical diagnosis of TF. Patients admitted with other diagnosis of fever were excluded from the study. Before the study, the guardians of the patients were informed about the objectives of the study, confidentiality was maintained and written informed consent and written assent were taken. Data was collected using semi-structured proforma. Blood samples were collected using universal precautions and brain heart infusion which establishes the growth of pathogens causing bacteremia and septicemia was used as culture medium. Data was analyzed using the Statistical Package of Social version 11.5. Point estimate at 95% confidence interval and descriptive statistics were calculated.

## RESULTS

Among 7450 patients, 151 (2.03%) at 95% Confidence Interval (1.71-2.35) patients were diagnosed with enteric fever. Among them, 85 (56.29%) were male and 66 (43.71%) were female (Table 1).

**Table 1:** Baseline characteristics of the participants (n=151).

Age (years)	n (%)
<5	46 (30.46)
5-9	34 (22.52)
>/10	71 (47.02)
**Gender**	
Male	85 (56.29)
Female	66 (43.71)
**Treatment received before presenting to BPKIHS**	
Yes	142 (94.04)
No	9 (5.96)

Median year of presentation with inter-quartile range was 7.00 (1.60-14.00). Oldest patient with the diagnosis was 14 years and the youngest was 3 years old. The mean weight was 22.14±8.35, the heaviest one being 52kg and the lightest one 9kg weight.

Antibiotics followed by paracetamol and ibuprofen combination were the most common drugs taken by patient while presenting to the hospital (Table 2).

**Table 2:** Drugs received before presenting to BPKIHS (n=142).

Pre-hospitalization treatment	n (%)
Antibiotics	142 (100)
Azithromycin	54 (38.03)
Ceftriaxone	33 (23.24)
Cefixime	21 (14.79)
Amoxicillin	17 (11.97)
Ofloxacin	15 (10.56)
Paracetamol + Ibuprofen	65 (45.77)
Paracetamol	63 (44.37)
Fexofenadine	18 (12.68)

Fever was present in all patients presenting to the hospital followed by abdominal pain 94 (62.25%) and diarrhoea 73 (48.34%) (Table 3).

**Table 3:** Clinical feature of pediatric patients admitted for Typhoid fever in BPKIHS (n=151).

Clinical feature	n (%)
Fever	151 (100)
Abdominal pain	94 (62.25)
Headache	71 (47.02)
Diarrhoea	73 (48.34)
Constipation	12 (7.95)
Splenomegaly	10 (6.62)
Coated tongue	31 (20.52)
Hepatomegaly	15 (9.93)

The mean duration of fever was 5.63±3.23. At extremes, one patient had a fever duration of 3 days (minimum) and other had that of 16 days (maximum). The mean temperature was 102.1±0.25°F.Ceftriaxone was prescribed to all patients admitted as typhoid fever. Chloroquine was prescribed to 48 (71.70%) patients and Artesunate to 5 (3.31%) patients as they came from malaria endemic area, which were later withdrawn after ruling it out (Table 4).

**Table 4:** Empirical treatment prescribed before culture reports were received (n = 151).

Antibiotics	n (%)
Ceftriaxone	151 (100)
Paracetamol	127 (84.1)
Azithromycin	65 (43.05)
Lariago (Chloroquine)	48 (71.79)
Clindamycin	7 (4.64)
Artesunate	5 (3.31)
Amikacin	3 (1.99)
Metronidazole	4 (2.65)

Widal test was positive in only 11 (7.28%) patients, however, 140 (92.72%) patients had non-significant titre (<1:80) in the widal test. Ceftriaxone was the most commonly prescribed empirical antibiotic while ciprofloxacin was sensitive.

Only 14 (9.27%) patients were leucopenic and 23 (15.23%) patients had leukocytosis. The median and interquartile total blood count was 5.9x10^3^ (43x10^2^ - 124x10^2^) where maximum and minimum total blood count were 269x10^2^ and 27x10^2^ respectively. The median and interquartile platelet count was 213x10^3^ (12x10^4^-286x10^3^) respectively.

Two-third of the patients (96/151) was hospitalized for at least 6 days, with the longest hospital stay of 14 days and shortest of 3 days. Mean defervesecene period azithromycin and ceftriaxone combination was 6±1.38 days with maximum of 12 and minimum of 3 days respectively. Mean defervesecene period for ceftriaxone when used alone was 6±2.72 with maximum and minimum defervesecene period of 11 and 3 days respectively. All the admitted patients recovered with no complications.

## DISCUSSION

Typhoid fever is predominantly caused by serovars typhi and paratyphi of Salmonella enterica. A blood culture is required to establish the confirmatory diagnosis of TF.

This study showed the occurrence rate of typhoid fever in the pediatric age group of BPKIHS was 2.03% which was similar to the study done by Prajapati et al.^5^ The current study shows highest frequency in above 10 years of age whereas Prajapati et al.^5^ found the highest frequency among below 10 children in their study. This may be due to difference in location and cultural pattern and that the tween age groups at Terai belt are more independent from their parents and hence are more exposed to unsafe drinking water and improper hygiene. Male showed more predominance in this study than females which is in consistent to the study conducted by Gosai Mehul et al.^6^ which may be because male are more exposed to outdoor activities in comparison to female.^7^

Our study showed more than 3/4^th^ patients received treatment before reaching BPKIHS. This may be because of easy accessibility of medicine over the counter and fear of relatively higher expenditure for treatment in the hospital. The most common prehospital antibiotic received by patient before being admitted in BPKIHS was Azithromycin followed by Ceftriaxone.

Fever 100%, abdominal pain 62.25%, headache 47.02% and diarrhea 48.34% were the commonest presentation in this study which is in consistent to the study conducted in Dhulikhel hospital^8^ but in contradictory to the study conducted in Kathmandu^9^ which reports coated tongue, relative bradycardia and splenomegaly as the commonest presentation. Ceftriaxone was empirically in all admitted patients. This may be because third generation cephalosporin are bactericidal drugs with increased activity against gram negative bacteria with lesser toxicity and greater safety^10^ and have fever clearance time averaging one week.^11^ Serum positivity for Widal test and Blood culture is low in this study as compared to other studies^5^ which might be because of initiation of antibiotics prior to sample collection. Resistance to ampicillin and trimethoprim-sulfamethoxazole and no resistance to ceftriaxone was observed in this study however, taking defervescence period into consideration, this study sample did not prove superiority of ceftriaxone and azithromycin (mean defervescence period 6±1.38 days over ceftriaxone (mean deference period 6±2.72 days alone.

This study reveals that 2/3^rd^ of the patients had at least 6 days of hospital stay which is comparatively longer for quinolone group.^12^

Small sample size and short duration of study were the major limitation of this study.

## CONCLUSIONS

Occurrence rate of Typhoid Fever was similar to other studies. Azithromycin was the most common pre-hospital antibiotic and Ceftriaxone was the most common empirical inpatient antibiotic prescribed. As the majority of patients were treated with antibiotic prior to hospital visit, antibiotic susceptibility could not be well established. Therefore, based on these observations, a further surveillance on typhoid fever and the antimicrobial susceptibility pattern is recommended.

## References

[1] Geneva: World Health Organization. (2018). Typhoid [Internet].

[2] Bhutta ZA, Dewraj HL (2006). Current concepts in the diagnosis and treatment of typhoid fever.. BMJ..

[3] Petersiel N, Shresta S, Tamrakar R, Koju R, Madhup S, Shresta S (2018). The epidemiology of typhoid fever in the Dhulikhel area, Nepal: A prospective cohort study.. PLoS One..

[4] Woodward TE, Smadel JE (1948). Preliminary report on the beneficial effect of chloromycetin in the treatment of typhoid fever.. Ann Intern Med..

[5] Prajapati B, Rai GK, Rai SK (2008). Prevalence of Salmonella typhi and paratyphi infection in children: a hospital based study.. Nepal Med Coll J..

[6] Gosai Mehul M, Hariyani Hareshwaree B, Purohit Payai H, Momin Abeda G (2011). A Study of Clinical Profile of Multidrug Resistant Typhoid Fever In Children.. NJIRM.

[7] Pokharel P, Rai SK, Karki G, Katuwal A, Vitrakoti R, Shrestha SK (2009). Study of enteric fever and antibiogram of Salmonella isolates at a teaching hospital in Kathmandu Valley.. Nepal Med Coll J..

[8] Sharma N, Koju R, Karmacharya B, Tamang MD, Makaju R, Nepali N (2004). Typhoid fever in Dhulikhel hospital, Nepal.. Kathmandu Univ Med J (KUMJ)..

[9] Mathura KC, Chaudhary D, Simkhada R, Pradhan M, Shrestha P, Gurubacharya DL (2005). Study of clinical profile and antibiotic sensitivity pattern in culture positive typhoid fever cases.. Kathmandu Univ Med J (KUMJ)..

[10] Goodman Louis S (2011). Penicillin, cephalosporin and other beta-lactum antibiotics..

[11] Parry CM (2004). The treatment of multidrug-resistant and nalidixic acid-resistant typhoid fever in Viet Nam.. Trans R Soc Trop Med Hyg..

[12] Bajracharya BL, Baral MR, Shakya S, Tuladhar P, Paudel M, Acharya B (2006). Clinical profile and antibiotics response in typhoid fever.. Kathmandu Univ Med J (KUMJ)..

